# Unequal enforcement, unequal inference: rethinking how we define policy exposures

**DOI:** 10.1093/haschl/qxaf063

**Published:** 2025-03-28

**Authors:** Simone Wien, Ariana N Mora, Michael R Kramer

**Affiliations:** Department of Epidemiology, Rollins School of Public Health, Emory University, Atlanta, GA 30322, United States; Department of Epidemiology, Rollins School of Public Health, Emory University, Atlanta, GA 30322, United States; Emory University School of Medicine, Atlanta, GA 30322, United States; Center for Rural Health and Health Disparities, Mercer University School of Medicine, Macon, GA 31207, United States

**Keywords:** social policy, causal inference, consistency assumption, policy evaluation, population health sciences

## Abstract

Social policy is a powerful intervention that has the potential to reduce or widen inequities in population health. While studies estimating the causal effect of social policies on health are valuable to policy stakeholders, these studies frequently report unstratified estimates for the total population, even though differential enforcement by sub-unit populations and geographies is common. The analytical decision to report unstratified estimates assumes a single version of the social policy is implemented uniformly across populations; in the presence of biased implementation, these analyses can generate misleading results that impede meaningful policy evaluation. In this commentary, we highlight the importance of considering differential policy effects among subpopulations as a function of poorly defined policy exposure (ie, lack of causal consistency) rather than effect measure modification or mediation. Framing the issue as one of poorly defined policy exposure allows for critical disentangling of the explicit and implicit purposes of a policy.

## Introduction

Social policy is a powerful intervention that has the potential to reduce or widen inequities in population health, and thus causal effect estimation is valuable to stakeholders. Studies estimating the effect of social policy frequently report the “unstratified” policy effect at its highest population unit (ie, the effect of a state policy for the entire state population). While this unstratified estimate assumes the social policy is implemented uniformly across populations, differential enforcement by sub-unit populations is common.^1,2^ Policies rarely include *de jure* discriminatory language, but *de facto* enforcement practices often result in targeted enforcement due to social group identity (eg, race, class, gender identity).^3^ Reporting unstratified estimates in the presence of biased implementation by social group membership can generate misleading results and impede meaningful policy evaluation.^1^ Expanding on prior work, we highlight the importance of considering differential policy effects among subpopulations as a function of poorly defined policy exposure (ie, lack of causal consistency) rather than effect measure modification or mediation. Framing the issue as one of poorly defined policy exposure allows for critical disentangling of the explicit and implicit purposes of a policy.^1,2,4^

## What is the challenge?

Tennessee's 2014 “Fetal Assault Law” (SB1391) is an example of a general policy marked by socially biased enforcement. In response to rising rates of neonatal opioid withdrawal syndrome, this law criminalized substance use during pregnancy. Unsurprisingly, SB1391 was not enforced uniformly: throughout the law's duration, Shelby County, a majority Black county, had one of the lowest rates of neonatal opioid withdrawal syndrome before and during the law's enforcement, yet had one of the highest arrest rates under the law.^5-7^ Additional evidence suggests that Black and low-income rural communities experienced disproportionate levels of surveillance and enforcement.^5,6^ Despite this, state-level estimates of the effect of SB1391 on health outcomes ignore different “versions” of enforcement as a result of race and rurality, violating the consistency assumption.^8,9^

**Figure 1.**
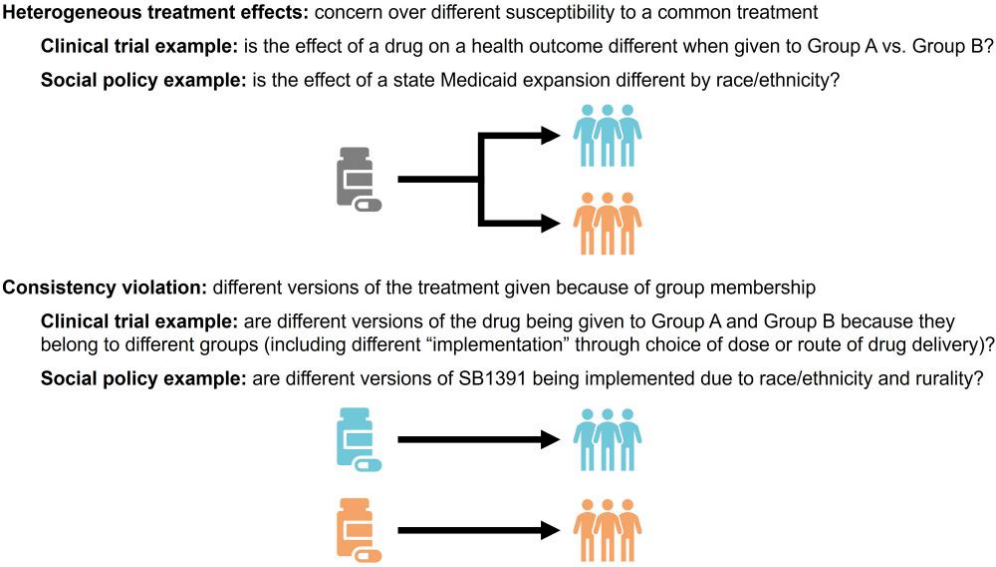
Distinction between HTEs and poorly defined exposures when estimating the causal effect of social policy.

We distinguish this scenario from heterogeneous treatment effects (HTEs) (ie, effect measure modification). HTEs are concerned with different susceptibility to a common treatment. Framing this in terms of a clinical trial estimating the effect of a drug, HTEs suggest a drug works differently when given to Group A vs Group B. A violation of the consistency assumption suggests qualitatively different versions of the drug (including differences in dosing or administration) are being given to Group A and Group B because they belong to different groups (Figure 1).^2^ While HTEs are common in social policy (eg, the impact of state Medicaid expansion on outcomes by race/ethnicity), in the case of SB1391, there is evidence that Black and/or low-income rural groups received different versions of SB1391 during its enforcement or “allocation” compared with other populations in the state.^6^

Framing the biased implementation of SB1391 as a consistency violation makes clear that estimating SB1391 for the entire population of Tennessee is problematic, as it would not be a well-defined policy exposure to say that Black and low-income rural groups experienced the same version of SB1391 as the rest of the state when there is evidence to the contrary. Said another way, consistency clearly articulates the problem as an overtly ill-defined intervention, rather than heterogeneous susceptibility of social groups experiencing it.

## What happens if this challenge is not addressed?

Ignoring biased implementation by reporting unstratified social policy effect estimates has analytical and theoretical implications.^1^ First, even when *de jure* policy language lacks overt discriminatory content, *de facto* policy implementation can be biased. Reproductive and substance use policies provide examples in which biased enforcement could be considered part of the policy itself rather than downstream factors or mediators.^3^ The prenatal drug testing policy at the center of the Supreme Court case *Fergusion* vs. *City of Charleston* (2001) is another example where biased implementation demonstrates covert intentions of the policy. Despite similar drug use recorded between Black and White patients and predetermined testing criteria, virtually all patients tested through the policy were Black, even though the policy as written implies all patients were subject to it.^3,6^ Defining this policy as a singular, well-defined intervention experienced by all patients both yields misleading estimates, as almost no White individuals experienced the policy, and obscures how this policy may have exacerbated health inequities by race.

Second, inappropriate confounder selection can occur, as confounder selection for the exposure-outcome relationship for one version of the policy may be incorrect for another version. For example, the set of potential confounders for the effect of SB1391 as experienced by individuals living in rural counties may include midwives per capita, whereas the set of potential confounders for SB1391 as experienced by individuals living in urban counties may include the availability of public transportation. Framing the effect of SB1391 at the state level in light of biased implementation as a violation of the consistency assumption allows us to specify confounders for each version of the policy, which typically is not considered for HTEs.

Third, failure to identify multiple versions of the treatment when they exist can result in misleading evidence in policy evaluation.^1^ For example, it is possible that when evaluating a policy at the state level, the effect may appear null, but in the setting of biased enforcement, the potentially harmful policy effect within sub-populations is masked due to aggregation. If only the aggregated null effect is reported and differential enforcement by social group is ignored, this policy estimate could lead stakeholders to spurious assumptions about its impact in sub-groups.

## What are potential strategies to address this challenge?

A general strategy to address poorly-defined exposures is to redefine the exposure such that it is “sufficiently well-defined” based on subject matter expertise and available information.^10^ We recommend the use of theory and data, particularly from legal sources, to classify policy exposures and to test multiple versions of policy.^1,2,4^ We make the additional recommendation that if variation in a social policy occurs due to social group membership, restricting or stratifying by that membership is appropriate and addresses consistency assumption violations. In the case of SB1391, local organizations and legal scholars provided evidence that Black and low-income rural communities in Tennessee received distinctly different versions of the policy due to their social group identities.^5,6^ Therefore, instead of generating one effect estimate for the state of Tennessee, operationalizing multiple versions of the policy by race and rurality more appropriately evaluates SB1391's health effects. For each “version” (eg, Black urban communities, White rural communities), investigators would define an exposed social group and appropriate comparison along with an estimation strategy to address relevant confounders.

## Conclusion

In the presence of biased enforcement as a result of social group membership, poorly defined social policy exposures are often evaluated through unstratified policy effect estimates. While social policy has the potential to repair existing health inequities, social policy evaluation that assumes a single version of the law—despite evidence of multiple enforcement regimes—obscures important implementation differences that affect vulnerable populations. Population health scientists should identify whether *de facto* policy implementation varies by social group and make appropriate analytical choices to highlight this policy variation. Without taking these considerations into account, non-stratified social policy evaluation may violate key causal assumptions, thus potentially lead to inaccurate and biased effect estimates, which can impede informed decision-making and further perpetuate existing inequities.

## Supplementary Material

qxaf063_Supplementary_Data
